# Mr BMT Achieves Systemic Macrophage Replacement With Preservation of Tissue Homeostasis

**DOI:** 10.1002/advs.202519669

**Published:** 2026-08-14

**Authors:** Yufei Xu, Miaozhan Zou, Yunshang Bai, Yang He, Zhihao Jin, Baozhi Yang, Jinjin Du, Pei Ouyang, Yuanyuan Cai, Yuheng Zhang, Bingying Du, Chengcheng Ge, Jian‐an Gong, Xiaoyu Li, Yuanyi Zheng, Jing Ding, Bo Peng, Yanxia Rao

**Affiliations:** ^1^ Department of Neurology, Zhongshan Hospital Laboratory Animal Center Institute for Translational Brain Research State Key Laboratory of Brain Function and Disorders MOE Frontiers Center for Brain Science Fudan SANS Neuroscience Center Fudan University Shanghai China; ^2^ Department of Neurosurgery, Huashan Hospital MOE Innovative Center for New Drug Development of Immune Inflammatory Diseases Shanghai Key Laboratory of Gene Editing and Cell Therapy for Rare Diseases Center for Clinical Neuro‐AI Fudan University Shanghai China; ^3^ National Children's Medical Center Children's Hospital Fudan University Shanghai China; ^4^ School of Basic Medical Sciences Jinzhou Medical University Jinzhou Liaoning China; ^5^ Department of Ultrasound in Medicine, Shanghai Sixth People's Hospital Shanghai Institute of Ultrasound in Medicine Shanghai Key Laboratory of Neuro‐Ultrasound for Diagnosis and Treatment Shanghai Jiao Tong University School of Medicine Shanghai China

**Keywords:** BMT, CSF1R, macrophage replacement, microglia replacement, Mr BMT

## Abstract

Microglia replacement is a novel and clinically validated therapeutic framework for brain diseases. Microglia replacement by bone marrow transplantation (Mr BMT) is among the most widely used strategies, achieving efficient replacement and robust therapeutic efficacy. However, Mr BMT affects not only the brain but also the peripheral system. In this study, we comprehensively investigated its effects on peripheral organs, including the liver, kidney, spleen, and lung. We found that Mr BMT achieved robust and sustained macrophage replacement across multiple organs. Although the replaced macrophages broadly acquired organ‐specific macrophage characteristics, they retained persistent molecular differences from naïve resident macrophages, suggesting that macrophage identity is simultaneously shaped by the local microenvironment and developmental ontogeny. Despite molecular remodeling to some extent, overall tissue architecture and biological function were preserved after Mr BMT. Together, our findings demonstrate that Mr BMT establishes durable systemic macrophage replacement in peripheral organs while preserving overt tissue homeostasis.

## Graphical abstract

Microglia replacement by bone marrow transplantation (Mr BMT) enables systemic replacement of tissue‐resident macrophages. Despite persistent macrophage and tissue remodeling across multiple organs, core biological functions and innate immune responses remain preserved, supporting long‐term maintenance of organismal homeostasis and the therapeutic potential of this strategy.

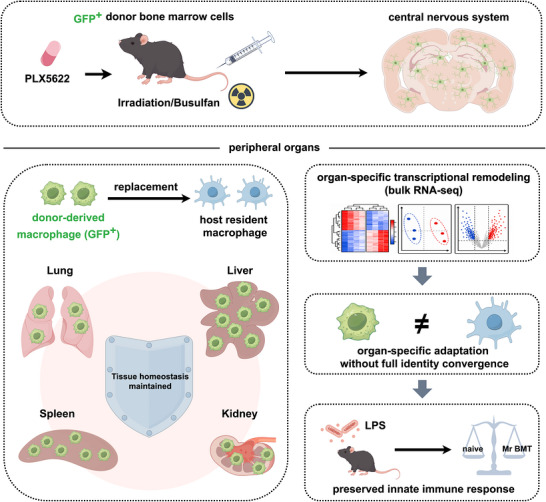

## 1 Introduction

Tissue‐resident macrophages, including microglia, the specialized macrophage of the brain parenchyma, serve as lifelong sentinels that continuously survey the microenvironment and eliminate pathogens [1]. Beyond immune surveillance, microglia and other tissue‐resident macrophages are indispensable for development, homeostasis, tissue repair, and host defense in both the central nervous system (CNS) and peripheral organs [2, 3]. Dysregulation or genetic mutations in these cells have been implicated in a wide range of disorders, including neurodegeneration, autoimmune diseases, chronic inflammation, and bone disorders [4]. To address conditions associated with macrophage dysfunction, traditional bone marrow transplantation (tBMT) has long been used to treat inherited immune disorders, such as severe combined immunodeficiency (SCID) [5], as well as autoimmune diseases [6].

However, tBMT shows a critical limitation in the context of CNS macrophage replacement: it achieves only minimal engraftment of donor‐derived cells into the brain, because long‐lived microglia are poorly replaced under homeostatic conditions. To overcome this limitation, we established efficient strategies for CNS‐wide microglia replacement [7, 8]. Microglia replacement by bone marrow transplantation (Mr BMT) is one of our microglia replacement strategies [7], and it has shown promise for modeling and potentially treating diseases including adult‐onset leukoencephalopathy with axonal spheroids and pigmented glia (ALSP) [9], Alzheimer's disease (AD) [7, 10], and Prosaposin deficiency [11]. Mr BMT involves depleting resident microglia to establish a microglia‐free microenvironment, followed by transplantation of donor bone marrow cells (BMCs) [12, 13]. CSF1R inhibitors, such as PLX5622, are widely used to deplete microglia in the CNS. The CSF1R signaling pathway is important for migration, function, and survival of macrophages, including both microglia and peripheral tissue macrophages [14]. As a result, perturbation of CSF1R signaling may influence the homeostasis in peripheral tissues. Recent studies have revealed that PLX5622 exerts effects beyond macrophages, including perturbations of endothelial function and disruption of hepatic cellular homeostasis [15, 16]. As an increasingly recognized and promising strategy for brain disease treatment, it is important to evaluate the long‐term safety and biological effects of Mr BMT on peripheral organs.

## 2 Results

### 2.1 CSF1R Inhibitor Ablates Peripheral Macrophages that Reach a Plateau After 2 Weeks

**FIGURE 1:**
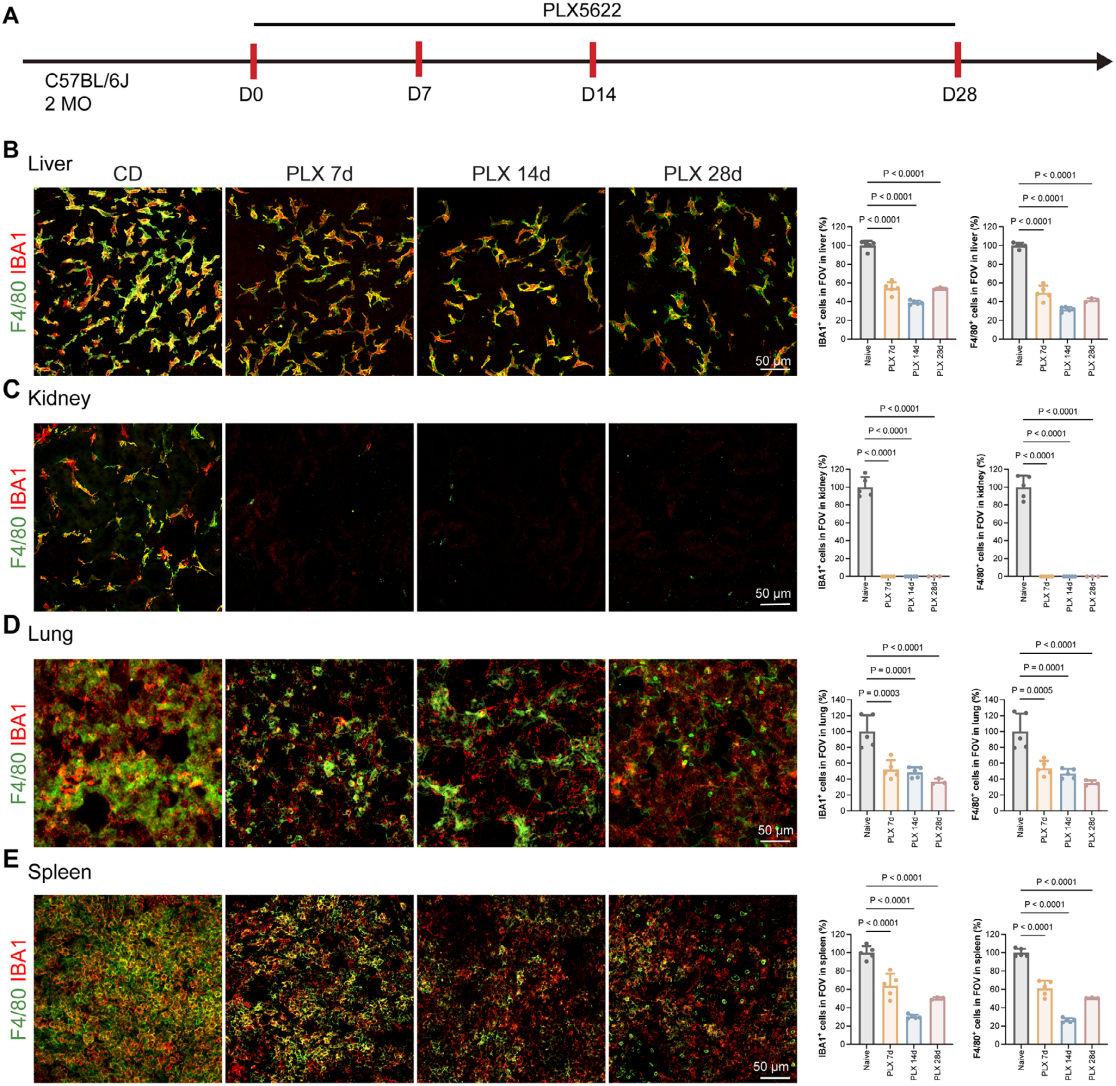
Dynamics of macrophage depletion across multiple tissues following PLX5622 administration. (A) Experimental design. (B–E) Representative confocal images and quantification showing the expression of IBA1 (red) and F4/80 (green) in the liver, kidney, lung, and spleen. N = 5 biological replicates for the naïve, PLX5622 7 d, and PLX5622 14 d groups; N = 3 biological replicates for the PLX5622 28 d group. Data are presented as mean ± SD. One‐way ANOVA with Tukey's multiple comparisons test was used for statistical analysis. FOV, field of view; D or d, day.

PLX5622 is a CSF1R antagonist that has been tested in different animal models [17, 18, 19]. To figure out the optimal time window for CSF1R inhibition that yields maximal macrophage depletion in peripheral tissues, we administered mice PLX5622 for 7, 14, and 28 days (Figure 1A). IBA1 and F4/80 are widely used as markers for microglia and macrophages, but they can also be detected on other immune cells [20, 21], raising the specificity concern when utilized in peripheral tissues. To address this concern, we simultaneously stained IBA1 with F4/80 (classical macrophage markers) and IBA1 with CD68 in the liver, kidney, spleen, and lung (Figure S1). CD68^+^ was nearly completely colocalized in IBA1^+^ cells in the liver, kidney, spleen, and lung. Similarly, more than 90% of F4/80^+^ cells were IBA1^+^ in the liver, kidney, and spleen, whereas the overlap in the lung was slightly lower (∼80%). These results reveal that IBA1 is a reliable marker for tissue‐resident macrophages in peripheral organs. We thus utilized IBA1 as the primary marker in subsequent analyses.

After PLX5622 administration for 7 days, the number of both IBA1^+^ and F4/80^+^ cells was significantly reduced in peripheral organs. Macrophage depletion in multiple tissues had reached a plateau at day 14, with efficiencies exceeding 60% in the liver, approximately 100% in the kidney, about 70% in the spleen, and approximately 50% in the lung. At day 28, macrophage cell numbers in the liver and spleen exhibited a slight increase (Figure 1B–E). These findings suggest that the extent of CSF1R inhibition‐mediated macrophage depletion differs across peripheral organs. This difference in depletion efficiency across organs most likely reflects the heterogeneity of macrophages rather than technical variability. A previous study has identified organ‐specific chromatin accessibility at the *Csf1r* locus [22]. The nearly complete depletion of kidney macrophages may reflect a stronger dependence on CSF1R signaling [22], whereas the lower depletion efficiency in the lung may result from reduced CSF1R dependency and compensatory survival signals (e.g., CSF2R) [22, 23]. Notably, PLX5622‐mediated depletion was less efficient in the liver, lung, and spleen than that in the CNS [9, 18, 19, 24, 25, 26] (Figure 1B–E and Figure S2). Together, these findings suggest that macrophage depletion efficiency upon CSF1R inhibition is highly tissue‐dependent and likely reflects differences in CSF1R dependency and local niche regulation.

### 2.2 Mr BMT Achieves Efficient and Durable Macrophage Replacement in Peripheral Organs

**FIGURE 2:**
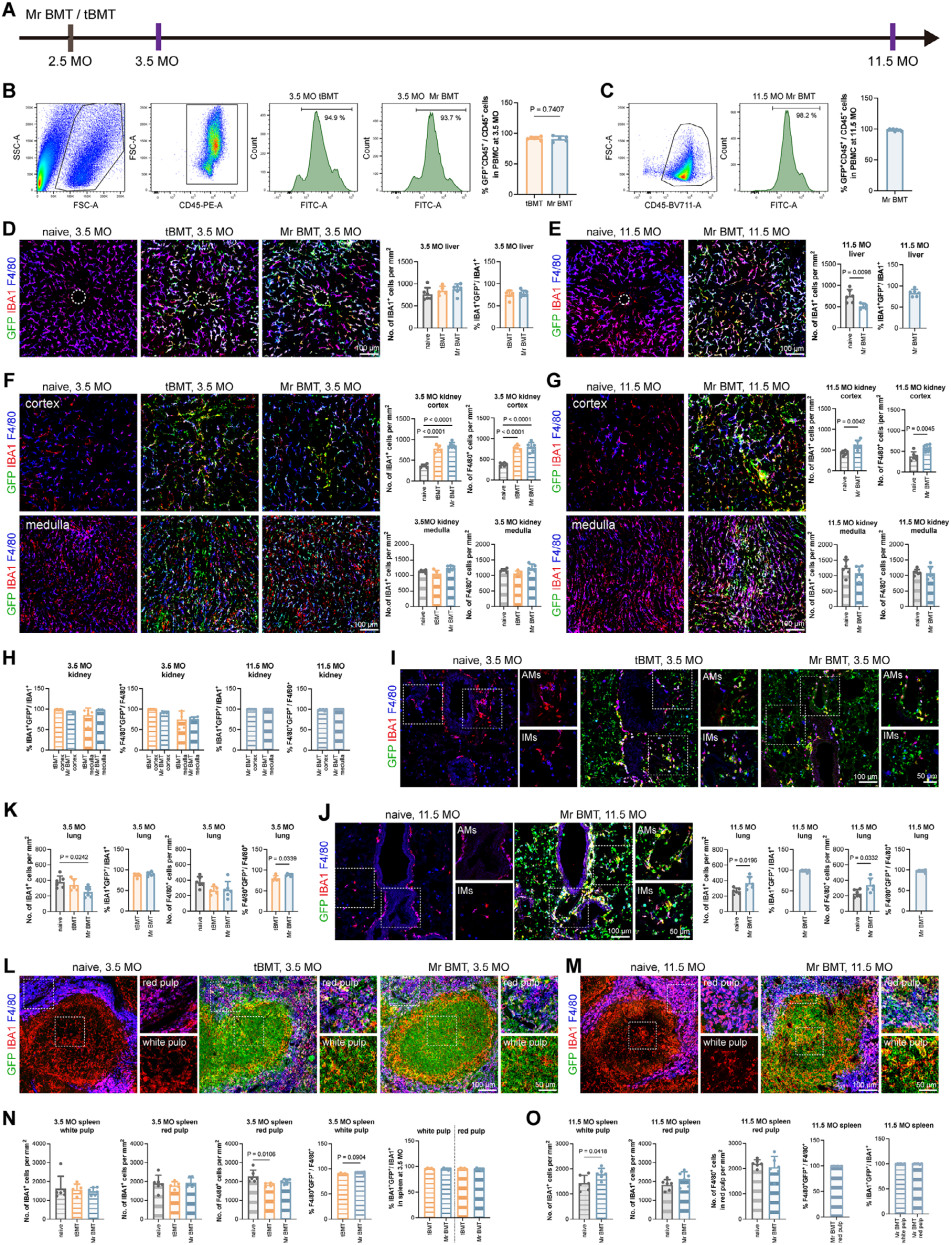
Long‐term engraftment of donor‐derived macrophages in peripheral organs following Mr BMT. (A) Experimental timeline. Timepoint for tBMT and Mr BMT in the scheme is defined as the time of BMC transplantation. (B–C) Representative flow cytometric plots and quantification of donor‐derived GFP^+^ cells in blood at 3.5 MO (B) and 11.5 MO (C). (D–O) Representative confocal images and quantitative analysis of BM‐derived cells (GFP, green) and macrophages (IBA1, red; F4/80, blue) in liver (D–E), kidney cortex and medulla (F–H), lung (I–K), and spleen (L–O) from naïve, tBMT, and Mr BMT groups at 3.5 MO and 11.5 MO. Insets indicate enlarged views of alveolar macrophages (AMs), interstitial macrophages (IMs), red pulp, and white pulp regions. N = 5–6 biological replicates per group for (B), N = 9 biological replicates for (C), and N = 5–6 biological replicates for (D–O). Data are presented as mean ± SD. Unpaired t‐test was used for panel (B) and all comparisons between naïve and Mr BMT groups at 11.5 MO, whereas one‐way ANOVA followed by Tukey's multiple comparisons test was used for comparisons among the naïve, tBMT, and Mr BMT groups at 3.5 MO (D–O). Only statistically significant comparisons (p < 0.05) are indicated. AMs, alveolar macrophages; IMs, interstitial macrophages; MO, month‐old; No., number.

We previously showed that a microglia‐free niche is necessary for efficient microglia replacement in the central nervous system [7, 8]. We thus tested whether macrophage depletion by CSF1R inhibition would enhance the replacement efficiency of peripheral tissue macrophages. To this end, we transplanted BM cells from Cx3cr1^+/GFP^ mice into recipients preconditioned with lethal irradiation, 9 Gy X‐ray, and assessed macrophage engraftment in the liver, kidney, lung, and spleen (Figure 2A).

At 1‐month post‐transplantation, we first assessed the level of donor chimerism by flow cytometry. Both tBMT and Mr BMT achieved robust donor chimerism, reaching 91.7% and 90.9% in the blood, respectively (Figure 2B). In the liver, approximately 75% of liver macrophages (GFP^+^IBA1^+^/IBA1^+^) were replaced by GFP^+^ donor‐derived macrophages (Figure 2D). Similarly, in the lung, over 90% of IBA1^+^ and F4/80^+^ resident macrophages were replaced by GFP^+^ donor‐derived cells (Figure 2I,K). In the kidney, approximately 97% of kidney macrophages were replaced, with donor‐derived cells evenly distributed both in the cortex and medulla (Figure 2F,H). Notably, the number of GFP^+^IBA1^+^ and GFP^+^F4/80^+^ cells was increased in the renal cortex after transplantation (Figure 2F). The spleen also exhibited high replacement efficiency, with nearly all resident spleen macrophages being replaced both in the white and red pulp (Figure 2L,N). We further tested the replacement efficiency by using busulfan, a more clinically relevant regimen for myeloablative conditioning, instead of lethal irradiation (Figure S3A). One month after transplantation, we observed a robust replacement of donor‐derived macrophages in the liver, kidney, lung, and spleen (Figure S3B–E). The levels of macrophage replacement were comparable to those observed with lethal irradiation, but busulfan provides a more clinically relevant framework. Collectively, our results demonstrate that donor‐derived macrophages can extensively repopulate resident macrophage compartments across multiple peripheral organs. Similar replacement efficiencies, both in Mr BMT and tBMT, were observed under lethal irradiation and high dosage of busulfan (Figure 2B–O and Figure S3B–E).

Previous studies have shown that macrophage replacement depends on niche availability in recipient tissues, where accessible niches allow the recruitment and differentiation of circulating progenitors [27, 28, 29]. When the niche availability is limited (e.g., under low‐dose irradiation), the engraftment efficiency is restricted by niche occupancy. In contrast, fully myeloablative conditions (e.g., 9 Gy X‐ray lethal irradiation or high‐dose busulfan) effectively deplete endogenous hematopoietic stem cells (HSCs) and dampen macrophages, thereby creating a sufficient niche space. We therefore reasoned that the comparable macrophage replacement efficiency in tBMT and Mr BMT under these conditions likely reflects a nearly complete niche saturation driven by systemic myeloablation. In this condition, the enhancement of replacement efficiency from the CSF1R inhibition‐mediated macrophage depletion is limited.

Finally, we examined the long‐term stability of engraftment and whether CSF1R had any lasting effects. Chimerism was measured in blood and peripheral tissues at 9 months after Mr BMT. Consistent with the observations at 1‐month, donor chimerism remained high, reaching 98% in blood (Figure 2C), and about 84% in liver, 99% in kidney, 92% in lung, and 99% in spleen (Figure 2E,G,H,J,M,O). Notably, macrophage density in the kidney cortex stayed elevated 9 months after transplantation (Figure 2G). We also found IBA1^+^ macrophage density in the splenic white pulp was increased at 9 months post‐Mr BMT (Figure 2O). Together, these results demonstrated that Mr BMT achieves durable macrophage replacement in peripheral organs.

### 2.3 Transcriptomic Alterations in Replaced Macrophages are Organ‐ and Stage‐Specific

**FIGURE 3:**
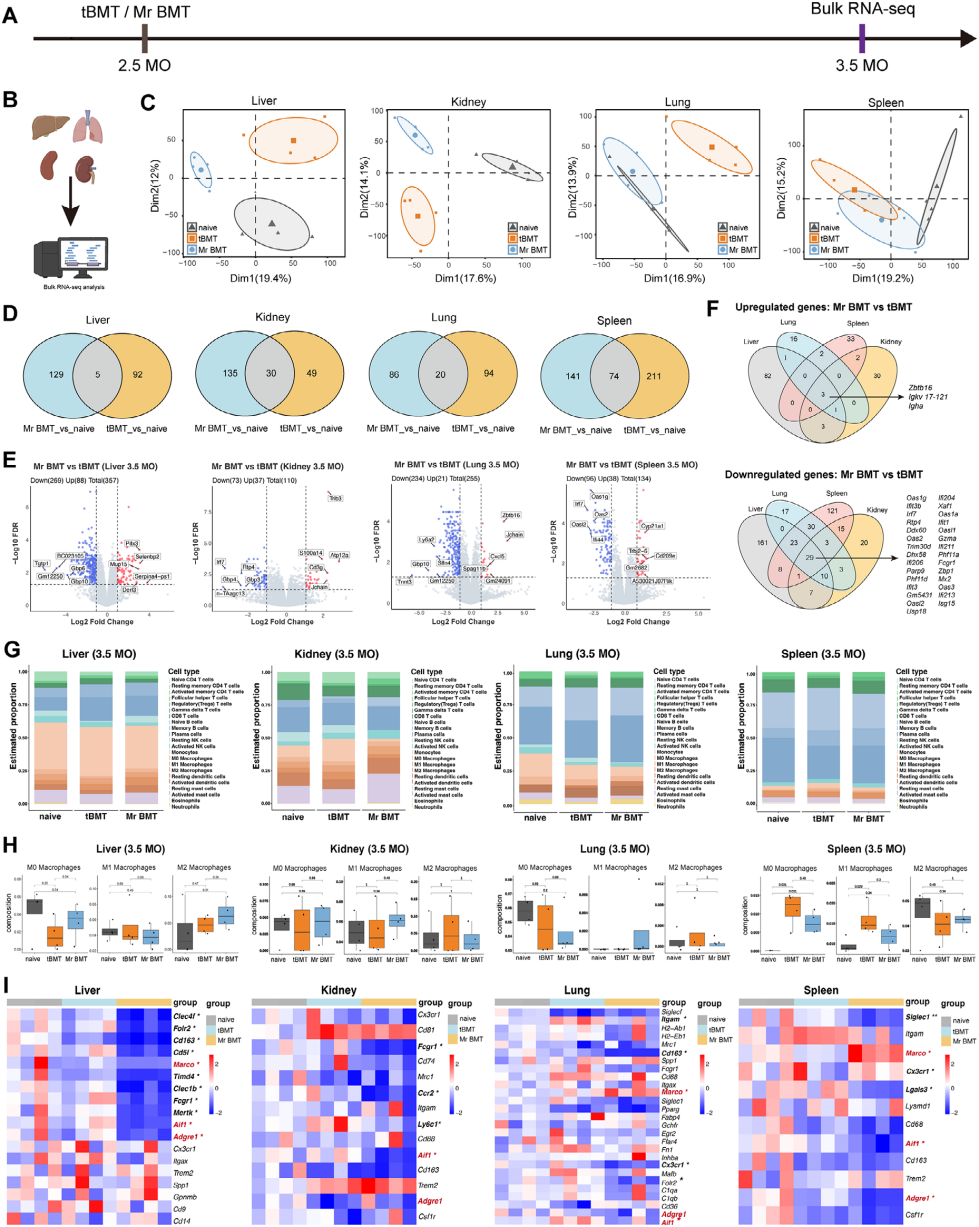
Donor‐derived macrophages acquire organ‐specific transcriptional programs following Mr BMT. (A) Experimental design. Timepoint for tBMT and Mr BMT in the scheme is defined as the time of BMC transplantation. (B) Schematic illustration of the bulk RNA‐seq analysis workflow in liver, kidney, lung, and spleen tissues. (C) Principal component analysis (PCA) of transcriptomic profiles from liver, kidney, lung, and spleen tissues in naïve, tBMT, and Mr BMT groups at 3.5 MO. (D) Venn diagrams showing the overlap of differentially expressed genes (DEGs) between Mr BMT versus naïve and tBMT versus naïve groups in liver, kidney, lung, and spleen tissues. (E–F) Volcano plots (E) and Venn diagrams (F) showing overlapping DEGs between Mr BMT and tBMT groups across organs at 3.5 MO. Representative genes are indicated. (G–H) CIBERSORT deconvolution analysis (G) and quantification of inferred macrophage subpopulations (H) across indicated organs from naïve, tBMT, and Mr BMT groups. (I) Heatmaps showing expression profiles of macrophage‐related genes in liver, kidney, lung, and spleen tissues among naïve, tBMT, and Mr BMT groups. Representative macrophage markers and tissue‐specific genes are highlighted. N = 4 biological replicates for each group. Data are presented as mean ± SD. Pairwise Wilcoxon rank‐sum tests were used for panel (H). Differentially expressed genes were identified using adjusted *p* < 0.05 and the indicated fold‐change cutoff. MO, month‐old.

Next, we asked whether additional PLX5622 treatment and macrophage replacement caused functional alterations in tissue homeostasis or immune responses. To this end, we performed RNA‐seq on the liver, kidney, lung, and spleen at 1 and 9 months after transplantation (Figure 3A,B). In the early stage of 1 month, principal component analysis (PCA) and Venn diagram analyses showed clear separation among naïve, Mr BMT, and tBMT groups in all four tissues, indicating that both tBMT and Mr BMT altered the global transcriptomic landscape (Figure 3C–F). To further explore which genes and functions were affected after Mr BMT and tBMT, we compared differentially expressed genes (DEGs) across these three groups (Figure S4). Heatmaps showed organ‐specific transcriptional changes. The Mr BMT group displayed distinct transcriptional patterns compared to both naïve and tBMT groups (Figure S4A–D). Both tBMT and Mr BMT shared immune‐related gene elevation across different tissues, including pro‐inflammatory cytokines and interferon‐responsive genes such as *Il1a*, *H2q1*, *Irf7*, and *Irf9* (Figure S4A–D). The RNA‐seq results were further confirmed by qPCR. Several inflammatory cytokines and chemokines, including *Il6*, *Tnf‐α*, *Il1b*, *Ccl3*, and *Ccl5*, were upregulated in peripheral organs after both tBMT and Mr BMT (Figure 5C). These observations indicate immune activation at the early stage after tBMT and Mr BMT.

To further understand the differences between Mr BMT and tBMT, we compared DEGs of Mr BMT vs tBMT across different organs. Only 3 genes were robustly upregulated in all peripheral organs we tested, including *Zbtb16*, *Igkv17‐121*, and *Igha*, suggesting alterations in plasma cell‐associated signatures after transplantation (Figure 3F). In contrast, 29 genes were robustly downregulated, including anti‐viral effectors (*Oas1g*, *Oas2*, *Oas3*, *Ifit1*, *Ifit3*, and *Ifit7*), interferon‐inducible genes (*Irf7*, *Isg15*, *Ifi204*, *Ifi206*, *Ifi211*, and *Ifi213*), and other immune regulators (*Dhx58*, *Trim30d*, and *Parp9*) (Figure 3E,F). These findings suggest a shared induction of lymphoid‐lineage cells, including T cell, B cell, and plasma cell‐associated genes, along with a broad suppression of interferon‐stimulated genes across peripheral tissues in Mr BMT relative to tBMT.

Given that these shared DEGs were strongly enriched in immune‐related pathways and linked to multiple immune cell populations, we therefore asked whether these transcriptional changes reflected alterations in immune cell composition or cell state. CIBERSORT deconvolution analysis detected major immune populations, including macrophages, monocytes, T cells, B cells, and plasma cells, across all tissues and experimental conditions (Figure 3G). Lymphoid cells accounted for approximately half of the immune signal in most tissues, and exceeded 80% in the spleen (Figure 3G). In non‐lymphoid tissues, this relatively high lymphoid proportion should be interpreted cautiously, as the residual circulating leukocytes due to incomplete perfusion may contribute to the bulk tissue transcriptome. Importantly, the overall composition of macrophage subsets, including M0‐like, M1‐like, and M2‐like macrophages, was broadly similar among naïve, tBMT, and Mr BMT groups (Figure 3H), implying that the broad immune‐cell compositions are not the major driver of the transcription shifts. In contrast, in most tissues, the expression of general‐macrophage signature genes, *Aif1, Adgre1*, as well as tissue‐specific macrophage signature genes, including *Timd4*, *Clec4f, Cd163, Marco*, and *Folr2*, were significantly downregulated in the Mr BMT condition, with the strongest effects observed in the liver, but not in tBMT (Figure 3I). However, the number of IBA1^+^ cells in most tissues from Mr BMT mice was comparable to or higher than that in naïve mice (Figure 2D–O). Collectively, these findings indicate that the CSF1R inhibition in Mr BMT induces a delayed macrophage characteristics acquisition, rather than cell number alteration at the early stage after transplantation.

**FIGURE 4:**
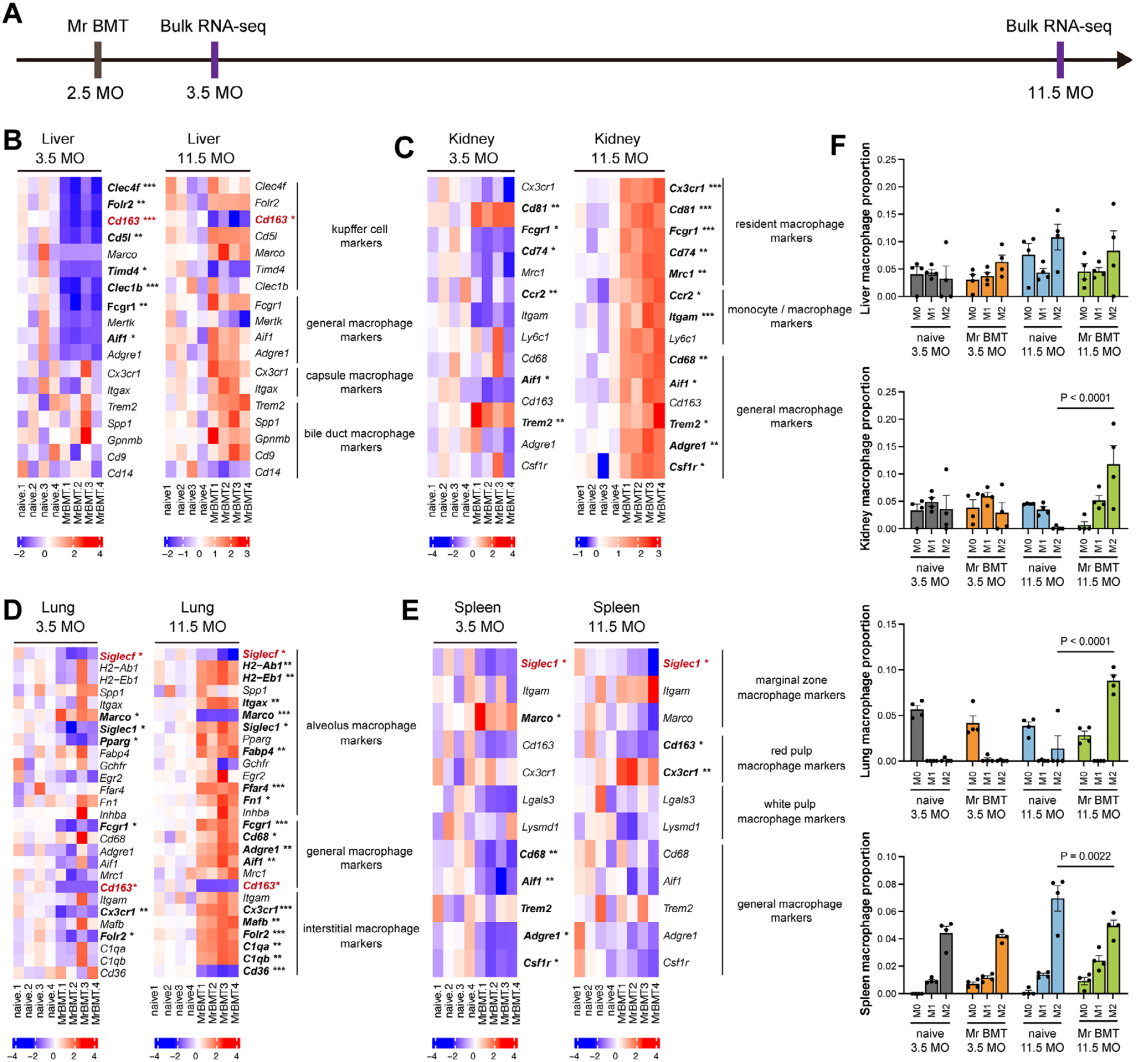
Donor‐derived macrophages exhibit persistent transcriptional differences from embryonically derived resident macrophages after Mr BMT. (A) Experimental timeline. Timepoint for Mr BMT in the scheme is defined as the time of BMC transplantation. (B–E) Heatmaps showing normalized expression (z‐score) of macrophage subtype‐associated marker genes in liver (B), kidney (C), lung (D), and spleen (E) at 3.5 MO and 11.5 MO in naïve and Mr BMT groups. Representative tissue‐resident macrophage signature genes are highlighted. (F) Quantification of macrophage subtype proportions in liver, kidney, lung, and spleen at 3.5 and 11.5 MO in naïve and Mr BMT groups. N = 4 biological replicates for each group. Data are presented as mean ± SD. One‐way ANOVA with Tukey's multiple comparisons test was used for panel (F). MO, month‐old.

Next, we examined the long‐term effects of Mr BMT on peripheral macrophage polarization and signature gene expression (Figure 4A). Histological analysis revealed organ‐specific changes in macrophage cell number at 9 months after transplantation. Macrophage density was decreased in the liver, but increased in the lung, kidney cortex, and splenic white pulp (Figure 2E,G,H,J,M,O). In line with the cell number, the macrophage signature genes *Aif1* (encoding IBA1) and *Adgre1* (encoding F4/80) were upregulated in the kidney and lung (Figure 4C,D). In the liver and spleen, their expression level in Mr BMT was comparable to those in naïve controls (Figure 4B–E). Notably, macrophage cell number and signature gene expression were not always concordant across tissues. The marker gene expression may also reflect macrophage status, including proliferation and polarization, rather than cell number alone. Indeed, transcriptomic profiling revealed tissue‐specific remodeling of CIBERSORT inferred macrophage subtypes after Mr BMT. In the kidney and lung, M0‐like macrophages were reduced while M2‐like macrophages were increased (Figure 4F). In contrast, M2‐like macrophages were reduced in the spleen while M1‐like macrophages were increased (Figure 4F).

Together, our results suggest that the PLX5622 administration in Mr BMT delays donor‐derived cells to acquire tissue‐resident macrophage transcriptional characteristics in the first month after transplantation. However, most macrophage signature genes gradually recovered to or even exceeded naïve levels over time, suggesting a progressive adaptation of donor‐derived macrophages to the local tissue microenvironment after Mr BMT.

### 2.4 BMT Induces a Long‐Term and Organ‐Specific Transcriptional Remodeling Across Peripheral Tissues

In addition to the organ‐specific macrophage remodeling, only a limited number of DEGs overlapped between early and late stages of Mr BMT (Figure S5). At 1‐month post‐Mr BMT, we observed a set of transcripts that were consistently dysregulated across tissues, including *Cdkn1a*, *Phlda3*, *Gdf15*, *Lcn2*, *Havcr1*, *Tff3*, *Psrc1*, *Gtse1*, *Ube2c*, and *Cdc20* (Figure S5). These genes have been previously reported to be strongly associated with cellular stress response, epithelial injury, tissue repair, as well as immune activation [30, 31, 32, 33]. By 9 months post‐transplantation, the sustained elevation of genes, including *Cdkn1a*, *Phlda3*, *Psrc1*, and *Gtse1* across tissues suggested the presence of persistent stress response and tissue repair after transplantation [30, 31] (Figure S5). Notably, several genes, particularly *Cdkn1a*, *Gdf15*, and *Phlda3*, have been reported as irradiation‐responsive genes [34], raising the possibility that irradiation conditioning may contribute to the persistent stress response and repair program we observed. Moreover, each tissue showed its own remodeling pattern. For instance, in the liver, *Cdkn1a*, *Phlda3*, *Tff3*, and *Lrtm1* remained elevated, while *Cd163* was suppressed, suggesting a sustained low‐grade stress state and incomplete restoration of the Kupffer cell (KC) identity in the liver [35] (Figure S5C). In the kidney, *Havcr1*/*Kim1*, *Lcn2*, complement component‐related genes, and chemokine genes were persistently elevated (Figure S5E), implying chronic inflammatory and fibrotic remodeling [31]. In the lung, sustained upregulation of mast cell‐associated genes (*Mcpt4, Tpsb2*, *Cpa3*, *Fcer1a*) [36], interferon‐stimulated genes, and chemokine genes suggested chronic immune activation (Figure S5G). In the spleen, *Slc4a1, Ank1*, *Spta1*, *Gypa*, *Ahsp*, *Epb42*, and *Tmod1* (Figure S5I), remained upregulated, indicating transplantation induces ongoing extramedullary erythropoiesis in the spleen [37, 38].

### 2.5 Mr BMT Preserves Tissue Innate Immune Responses Following LPS Challenge

**FIGURE 5:**
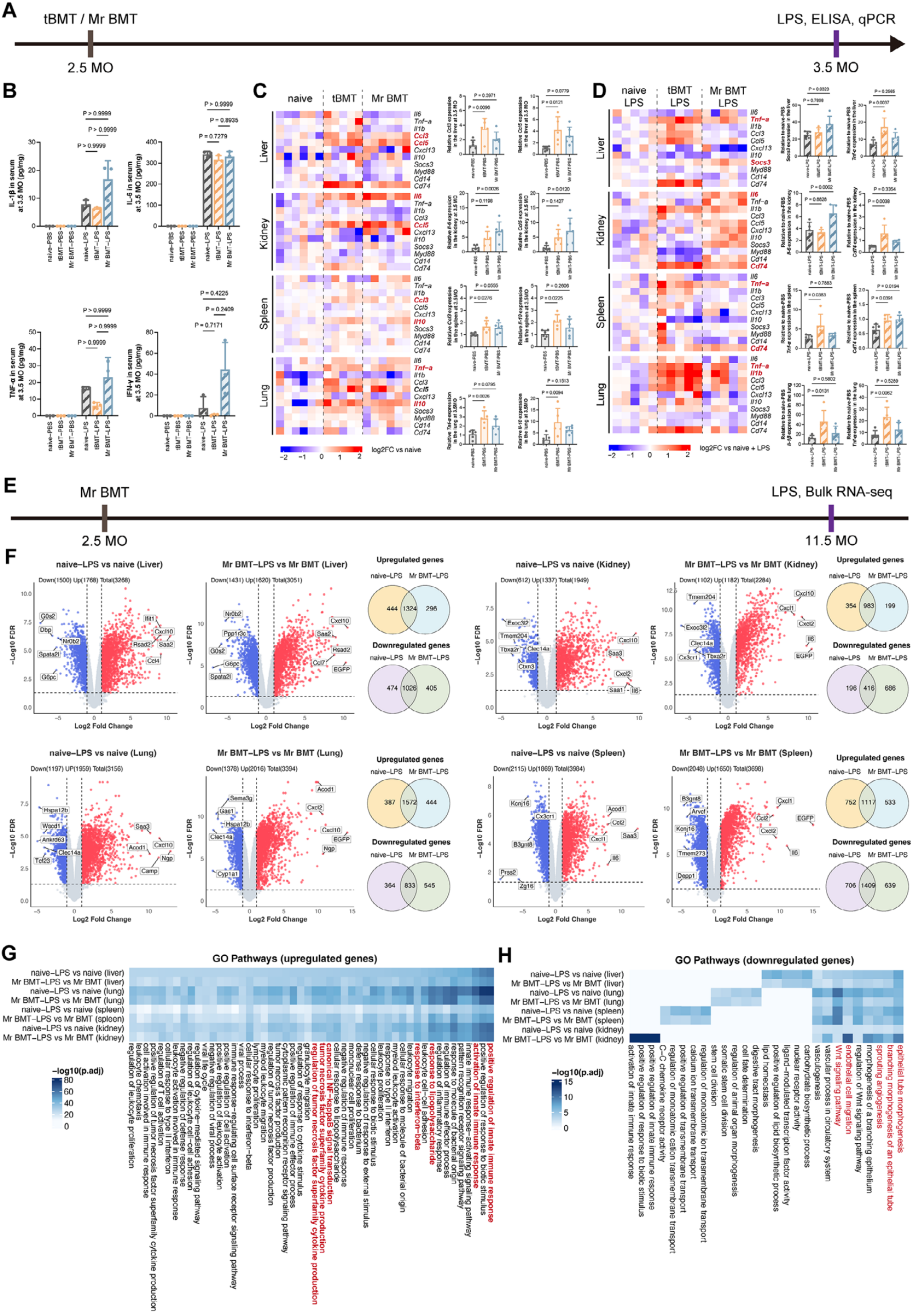
Tissues preserve systemic immune responses following LPS challenge after BMT. (A) Experimental timeline. Timepoint for tBMT and Mr BMT in the scheme is defined as the time of BMC transplantation. (B) Serum levels of IL‐1β, IL6, TNF‐α and IFN‐γ measured by ELISA in naïve, tBMT and Mr BMT mice following LPS treatment at 3.5 MO. (C,D) Heatmaps showing indicated immune response‐related genes expression measured by qPCR across organs from naïve, tBMT, and Mr BMT groups treated with PBS (C) or LPS (D). Bar plots showing selected genes expression. (E) Experimental timeline. Timepoint for Mr BMT in the scheme is defined as the time of BMC transplantation. (F) Volcano plots and Venn diagrams showing DEGs following LPS challenge in naïve and Mr BMT mice at 11.5 MO. (G,H) GO enrichment analysis of upregulated (G) and downregulated (H) genes across tissues following LPS treatment in the naïve and Mr BMT groups. N = 3 biological replicates for panel (B), N = 5 or 6 biological replicates for each group in (C,D), N = 4 biological replicates in (F–H). Data are presented as mean ± SD. One‐way ANOVA with Tukey's multiple comparisons test was used for panels (C,D), while Kruskal–Wallis test was used for panel (B). MO, month‐old.

To determine whether the early and late transcriptional remodeling observed after transplantation had functional consequences, we challenged the mice with lipopolysaccharide (LPS) (Figure 5A), which induces an intensive innate immune activation and barrier impairment in peripheral organs [39, 40, 41, 42, 43].

We first examined whether additional PLX5622 treatment used in Mr BMT influenced tissue response to LPS challenge. Following LPS challenge, both transplantation paradigms exhibited a robust inflammatory reaction, with an increase in circulating IL6, TNF‐α, IL‐1β, and IFN‐γ (Figure 5B). Mr BMT mice tended to have higher levels of TNF‐α, IL‐1β, and IFN‐γ than naïve and tBMT mice, but these differences were not statistically significant (Figure 5B). qPCR analysis confirmed that LPS induced robust activation of LPS‐responsive pathways across multiple peripheral organs, with significant upregulation of *Cd14* and *Myd88*, two core components of TLR signaling axis [44], together with strong induction of *Il6*, *Tnf*, and *Il1b* [45] (Figure S6A–D). Overall, Mr BMT and tBMT displayed comparable response profiles following LPS stimulation (Figure 5D). However, compared with naïve mice, both transplantation programs exhibited stronger induction of pro‐inflammatory cytokines expression, such as *Il6*, *Tnf, Il1b*, across tissues (Figure 5D). A possible explanation is that the pre‐existing immune‐activated state observed in multiple tissues during the early recovery period (Figure 3D–F and Figure 5C) could prime peripheral tissues for enhanced responsiveness to second stimulation.

Next, we asked whether macrophage replacement by Mr BMT impaired the global tissue immune response to LPS challenge at the late stage (Figure 5E). RNA‐seq revealed LPS induced extensive transcriptional alterations in naïve and Mr BMT mice, with upregulated and downregulated genes collectively comprising over half of DEGs in each tissue (Figure 5F and Figure S6E–J). The overall direction and magnitude of response‐associated genes were highly similar between Mr BMT and naïve groups (Figure 5F and Figure S6G–J). Consistently, GO enrichment analysis confirmed strong activation of innate immune pathways across tissues, including response to LPS or bacterial organisms, canonical NF‐κB signaling, inflammatory cytokine production, IFN/TNF superfamily cytokine signaling, along with epithelial damage and Wnt signaling pathway disruption (Figure 5G,H). Together, these findings suggest that despite extensive macrophage replacement, Mr BMT preserved the intrinsic inflammatory responsiveness of peripheral tissues to acute immune challenge.

### 2.6 Bone Marrow‐Derived Macrophages Incompletely Acquire Kupffer Cell Identity in the Liver

Tissue‐resident macrophages in peripheral organs are seeded during embryogenesis and maintained by self‐renewal, with minimal input from adult hematopoiesis under steady‐state conditions [3, 46]. Previous studies have reported that BM‐derived cells only partially recapitulate the transcriptional, phenotypic, and functional features of bona fide embryonically derived resident macrophages [47, 48, 49, 50]. Consistent with this framework, we observed persistent downregulation of resident macrophage marker genes across organs, such as *Cd163* in the liver, *Siglecf* in the lung and spleen (Figure 4B–E).

**FIGURE 6:**
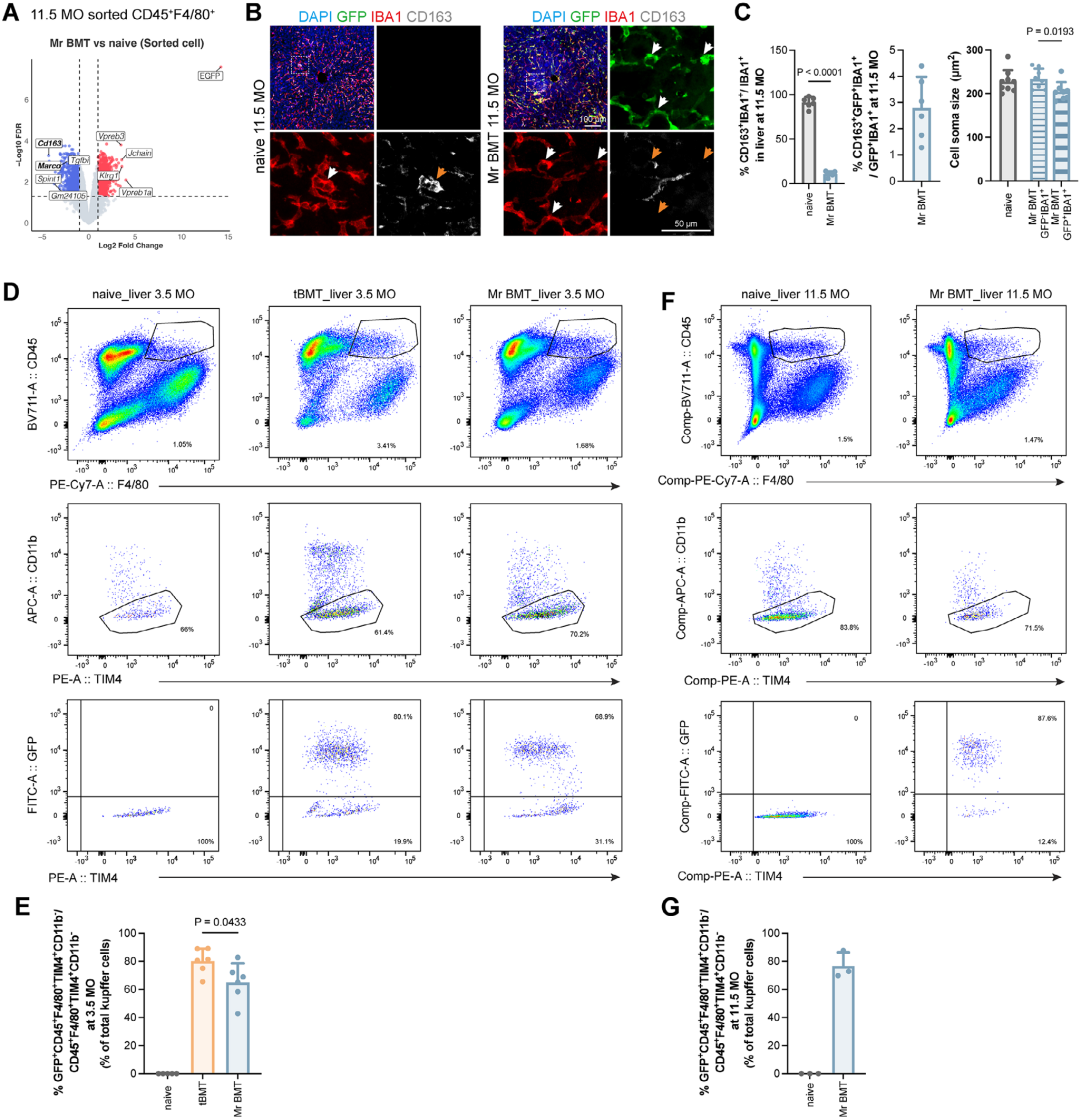
Donor‐derived macrophages acquire KC identity but retain persistent differences from resident KC in the liver. (A) Volcano plot showing DEGs in sorted CD45^+^F4/80^+^ and GFP^+^CD45^+^F4/80^+^ cells from naïve and Mr BMT groups at 11.5 MO. (B–C) Representative confocal images (B) and quantification (C) of GFP^+^ donor‐derived macrophages (IBA1, red) and CD163 (gray) expression in liver tissues from naïve and Mr BMT mice at 11.5 MO. (D–G) Representative flow cytometric plots (D, F) and quantification (E, G) of GFP^+^CD45^+^F4/80^+^CD11b^−^ cells in liver at 3.5 MO (D–E) and 11.5 MO (F–G) following tBMT and Mr BMT. N = 4 biological replicates for panel (A), N = 6–9 biological replicates for panel (C), N = 5–6 biological replicates per group for panel (E), and N = 3 biological replicates per group for panel (G). Data are presented as mean ± SD. Unpaired t‐test was used for panels (C), whereas one‐way ANOVA followed by Tukey's multiple comparisons test was used for panel (E). MO, month‐old.

Consistent with these findings, we observed a reduced expression of *CD163* and *Marco* in sorted CD45^+^F4/80^+^GFP^+^ cells in the liver at 9 months post‐transplantation (Figure 6A). Consistently, immunohistochemical analyses revealed a robust reduction in CD163 expression in the liver; only a minority of GFP^+^ cells (less than 7%) were double positive for IBA1 and CD163 in both tBMT and Mr BMT (Figure 6B,C and Figure S7A,B). In addition, BM‐derived IBA1^+^ cells exhibited a smaller body size compared to resident KCs (Figure 6C). However, flow cytometry analysis revealed that about 66% of the KCs population, displaying CD45^+^F4/80^+^TIM4^+^CD11b^–^ phenotype, in Mr BMT mice were donor‐derived (Figure 6D,E). The proportion was increased to 77% by 9 months post‐transplantation (Figure 6F,G). These observations suggested that BM‐derived cells underwent substantial adaptation to the hepatic niche but fail to fully acquire the embryonically derived KCs transcriptional program, even long‐term after transplantation. Notably, although the CD163 expression in donor‐derived liver macrophages was significantly reduced by both tBMT and Mr BMT, its level was higher in the tBMT group than in the Mr BMT group at 1 month after BMT (Figure 3I, and Figure S7A,B). This difference might be attributable to the CSF1R inhibition.

A previous study has demonstrated that yolk sac progenitors, fetal liver monocytes, and adult monocytes can efficiently engraft the lung and differentiate into functional lung macrophages [27]. This is particularly true for alveolar macrophages (AMs). Characteristics of AMs are strongly shaped by their microenvironmental signals, including GM‐CSF and TGF‐β, allowing substantial plasticity in their developmental origin [27]. In line with that, we observed that more than 60% of AMs (CD45^+^F4/80^+^CD11c^+^CD11b^–^) were donor‐derived replaced macrophages (Figure S7C–E), whereas more than 98% of interstitial macrophages (IMs) (CD45^+^F4/80^+^CD11c^–^CD11b^+^) were donor‐derived replaced macrophages (Figure S7C–E).

### 2.7 BM‐Derived Macrophages in the Liver Retain Core Macrophage Chromatin Programs but Fail to Fully Acquire Kupffer Cell Program

**FIGURE 7:**
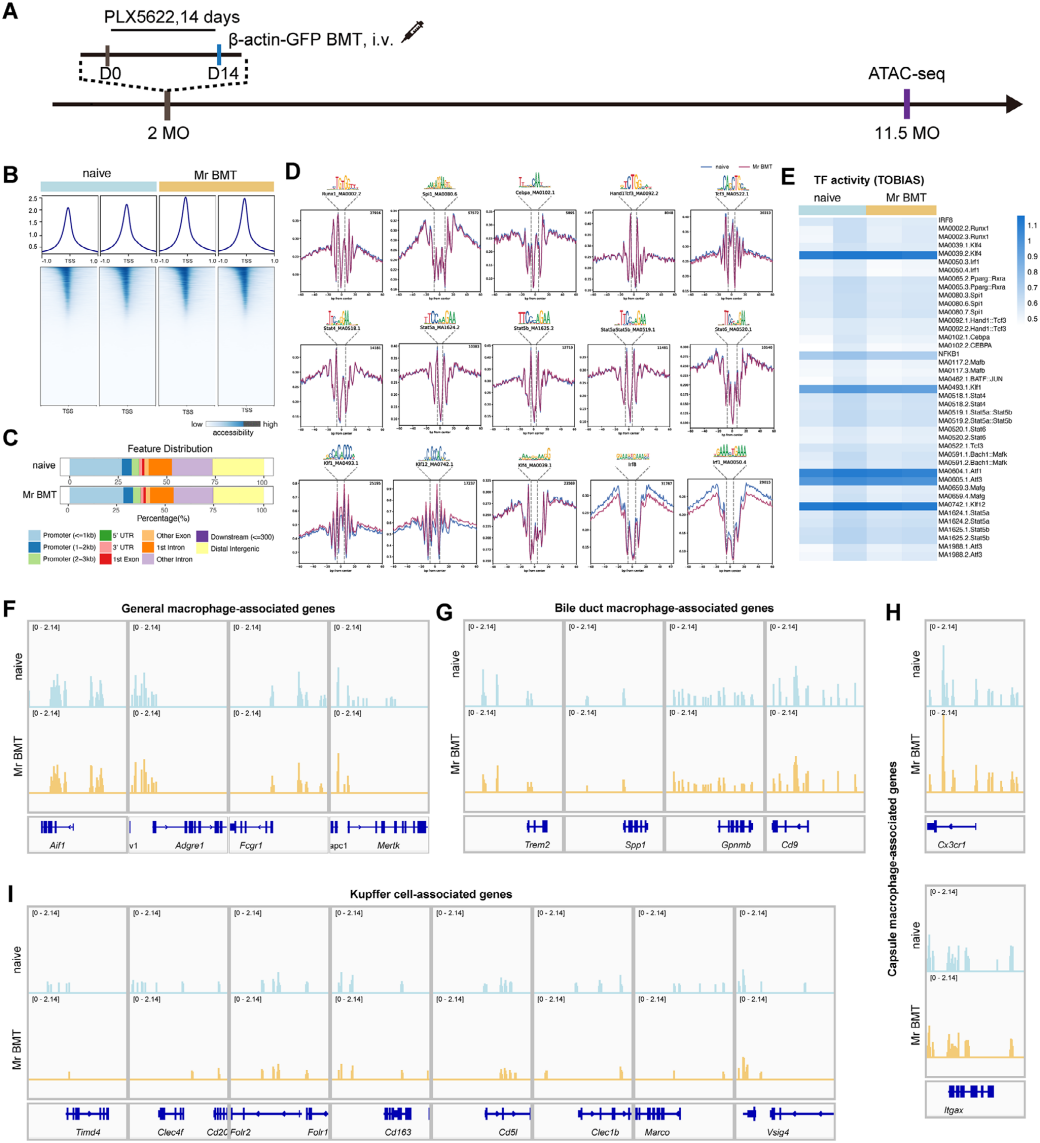
ATAC‐seq reveals distinct chromatin landscape underlying the incomplete acquisition of KC identity by donor‐derived liver macrophages. (A) Experimental design. Timepoint for Mr BMT in the scheme is defined as the time of PLX5622 treatment. (B) Read density profiles and heatmaps showing global ATAC‐seq signals centered around transcription start sites (TSS ± 1 kb) in sorted CD45^+^ and CD45^+^GFP^+^ cells following Mr BMT. (C) Genomic feature distribution of ATAC‐seq peaks in naïve and Mr BMT groups. (D,E) Footprint analysis of macrophage lineage‐determining and liver‐identity transcription factors (D) and corresponding transcription factor activity determined by TOBIAS analysis (E). (F–I) Representative ATAC‐seq tracks showing chromatin accessibility at loci associated with general macrophage‐associated genes (F), bile duct macrophage‐associated genes (G), capsule macrophage‐associated genes (H), and Kupffer cell‐associated genes (I). MO, month‐old.

To further characterize the BM‐derived liver macrophages, we performed ATAC‐seq on isolated liver CD45^+^ and CD45^+^GFP^+^ cells at 9 months post‐Mr BMT (Figure 7A). Globally, the accessible chromatin regions in both BM‐derived and embryonically derived cells were mainly enriched within ± 1 kilobase (kb) of the transcription start site (TSS) (Figure 7B). Compared to embryonically derived cells, BM‐derived cells showed increased accessibility around TSS‐proximal regions, while the overall distribution of ATAC peaks across flanking regulatory regions remained broadly similar, indicating preservation of a shared core macrophage chromatin architecture (Figure 7C). Motif analysis further uncovered enrichment of canonical macrophage lineage‐determining transcription factors (TFs), such as RUNX, PU.1/SPI1, C/EBP, STAT family members, KLF family and IRF family members [51, 52], and liver macrophage identity‐associated TFs, such as IRF8 [53] (Figure 7D). BM‐derived cells and embryonically derived cells displayed largely similar motif enrichment patterns (Figure 7E). Consistent with this shared motif distribution, we found that loci associated with general macrophage genes, including *Aif1*, *Adgre1/*F4/80, *Fcgr1a*/Cd64 and *Mertk* [52] (Figure 7F), bile duct macrophage‐associated genes including *Trem2*, *Spp1*, *Gpnmb*, and *Cd9* [52] (Figure 7G) as well as liver capsule macrophage‐associated genes *Cx3cr1*, *Itgax*/Cd11c (Figure 7H) exhibited similar chromatin accessibility in naïve and Mr BMT mice. In contrast, canonical KC‐associated genes [52], including *Timd4*, *Clec4f, Folr2, Cd163*, *Cd5l*, *Vsig4*, and *Marco*, showed decreased chromatin accessibility in BM‐derived cells (Figure 7I). Together, these findings suggest that BM‐derived macrophages acquire a broadly conserved liver macrophage regulatory program but fail to fully establish the mature KC chromatin landscape, consistent with our flow cytometric and immunofluorescence observations.

### 2.8 Mr BMT did not Impair Systemic Homeostasis or Central Nervous System Function

Given that we observed that Mr BMT exhibited an obvious reduction in tissue‐resident macrophage identity genes expression and a distinct transcriptional profile compared to tBMT and naïve mice during the early post‐transplantation period, we hypothesized that the additional PLX5622 conditioning used in the Mr BMT paradigm might interfere with tissue recovery following irradiation‐ or BU‐associated stress. Besides, recent studies indicate that PLX5622 exerts effects beyond macrophage depletion, including alterations in endothelial metabolic programs and hepatic xenobiotic metabolism [15, 16]. We therefore examined whether Mr BMT led to systemic physiological or metabolic abnormalities.

**FIGURE 8:**
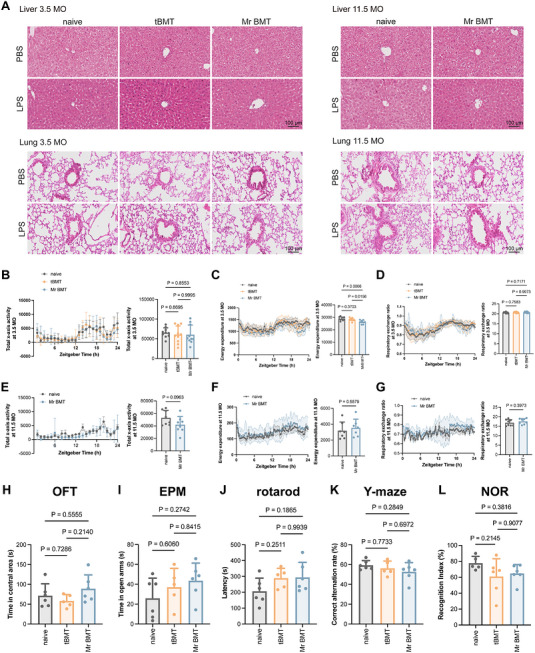
Mr BMT preserves long‐term physiological, metabolic and behavioral homeostasis. (A) Representative H&E staining images of the liver and lung from mice receiving PBS or LPS challenge in the indicated groups at 3.5 MO and 11.5 MO. (B–D) Metabolic cage analysis in naïve, tBMT, and Mr BMT mice at 3.5 MO, including total X‐axis activity (B), energy expenditure (C), and respiratory exchange ratio (RER) (D) across the light/dark cycle. (E–G) Metabolic cage analysis in naïve and Mr BMT mice at 11.5 MO, including total X‐axis activity (E), energy expenditure (F), and respiratory exchange ratio (RER) (G) across the light/dark cycle. (H–L) Behavioral tests including open field test (OFT, H), elevated plus maze (EPM, I), rotarod test (J), Y‐maze spontaneous alternation test (K), and novel object recognition test (NOR, L). N = 8–10 biological replicates for panel (B–D), N = 7–8 biological replicates for panel (E–G), N = 5–6 biological replicates for panel (H–L). Data are presented as mean ± SD. One‐way ANOVA with Tukey's multiple comparisons test was used for panels (B–D, H–L), unpaired t‐test was used for panels (E–G). MO, month‐old; s, second; h, hour.

Histological examination revealed no overt structural abnormalities in the liver or lung, under either steady state or after LPS challenge at the early stage after transplantation (Figure 8A). Even at 9 months after transplantation, no structure damage was detected in all tissues we examined, regardless of whether LPS challenged or not (Figure 8A). These findings indicate the integrity of peripheral tissues was overtly preserved after Mr BMT treatment.

To assess systemic metabolic homeostasis, we performed metabolic cage analysis at early and late stages after transplantation (Figure 8B–G). One month after transplantation, Mr BMT‐treated mice displayed a transient reduction in energy expenditure (Figure 8C). In contrast, the energy expenditure returned to baseline at 9 months after transplantation (Figure 8F), indicating the recovery of metabolic homeostasis over time. Serum biochemical analyses revealed that both transplantation paradigms caused an acute and transient reduction in serum alkaline phosphatase (ALP) levels at the early stage after transplantation (Figure S8A,C). The ALP level recovered at the late stage (Figure S8B,D). This early decrease is consistent with previous reports and likely reflected the suppressed osteoblastic activity and/or zinc deficiency after transplantation conditioning [54]. We observed a modest reduction in serum cholinesterase, high‐density lipoprotein (HDL), and total cholesterol (Figure S8D) at 9 months after transplantation, indicating a mild long‐term lipid metabolic alteration. Importantly, similar changes were also observed in tBMT [55]. Thus, these long‐term metabolic changes are not specific to Mr BMT but also tBMT, they appear to largely caused by BU or irradiation associated toxicity rather than macrophage replacement or PLX5622 treatment.

Finally, we examined the impact of Mr BMT on CNS function by conducting a battery of behavioral tests at 1‐month post‐transplantation. In open field test, the percentage of time spent in the central area, an indicator of anxiety‐like behavior, was comparable across all groups (Figure 8H). Similarly, in the elevated plus maze, the time spent in the open arms was comparable among groups, further indicating the absence of anxiety‐like behavior following BMT (Figure 8I). Cognitive performance assessed using the Y‐maze and novel object recognition (NOR) tests, also showed no difference among groups. Both Mr BMT and tBMT mice exhibited high spontaneous alternation percentages in Y‐maze (Figure 8K), and clear preference for the novel object in NOR (Figure 8L). Motor function, measured by rotarod test, showed no differences in latency to fall among groups (Figure 8J).

Together, these findings indicate that Mr BMT does not cause overt long‐term organ dysfunction or affect emotional, cognitive, or motor function, supporting the overall safety and tolerability of this transplantation strategy.

## 3 Discussion

In this study, we systematically examined the long‐term effects of Mr BMT on peripheral tissues. We observed that, under a fully myeloablative conditioning regimen, Mr BMT achieved donor‐cell engraftment efficiency comparable to that seen in tBMT. BM‐derived macrophages efficiently and persistently reconstituted macrophages in the liver, kidney, lung, and spleen. Although replaced macrophages remained transcriptionally and epigenetically distinct from the original tissue‐resident macrophages, these molecular differences did not result in overt functional impairment. The tissue integrity, overall metabolic state, and core biological functions were largely preserved following transplantation.

### 3.1 Macrophage Depletion Induces a Transient Effect on Peripheral Macrophage Engraftment

Previous studies reported that the macrophage‐free niche in peripheral organs enables monocyte‐ or BM‐derived macrophage engraftment [27, 29]. In this study, we showed that CSF1R inhibition successfully depleted macrophage across multiple tissues, thereby creating a macrophage‐free niche. In spite of that, we did not observe a higher engraftment efficiency by Mr BMT than that by tBMT. One possible explanation is that, either lethal irradiation or high‐dose BU alone already permits full donor HSCs engraftment, which override the niche‐opening benefits from macrophage depletion. This interpretation is supported by a recent study that PLX5622 treatment significantly improved macrophage engraftment in the liver under low‐dose irradiation, where donor cell engraftment is limited [28]. These findings suggest that although PLX5622‐mediated macrophage depletion shows limited benefit for peripheral macrophage replacement under highly myeloablative conditioning, macrophage depletion may still serve as an effective strategy to facilitate donor HSCs engraftment and reduce the requirement for high‐dosage irradiation or chemotherapy‐based conditioning.

On the other hand, additional macrophage depletion induced obvious alterations in macrophage transcriptional states compared with tBMT during the early post‐transplantation period. Across tissues, resident macrophage signature genes were broadly downregulated in Mr BMT mice relative to naïve and tBMT, particular in liver. In the liver, canonical macrophage and KC signature genes, such as *Aif1*, *Cd163*, *Timd4*, *Clec4f*, and *Marco*, were significantly downregulated. Consistent with these transcriptional changes, protein levels of CD163 and TIM4, which are two KC markers, were significantly reduced in Mr BMT compared with those in tBMT at the early stage after transplantation. These changes occurred despite preserved macrophage density and comparable engraftment efficiency between tBMT and Mr BMT in multiple tissues. This distinct phenotype suggests that PLX5622 did not impair macrophage engraftment or cause macrophage loss after transplantation. Rather, these findings suggest that PLX5622‐mediated macrophage depletion delays the acquisition of mature tissue macrophage molecular pattern by donor‐derived macrophages during early recovery phase. Importantly, most macrophage signature genes gradually returned to baseline levels or even exceeded them over time, implying that the early effects are largely transient. Recent studies have shown that macrophage depletion may impair hematopoietic recovery after HSC transplantation by disrupting HSCs homing, expansion, and niche support [56]. Therefore, it is possible that the delayed acquisition of mature macrophage characteristics observed in Mr BMT may partially reflect delayed hematopoietic reconstitution following macrophage depletion during the early recovery phase.

### 3.2 Bone Marrow Transplantation Induces Persistent Macrophage and Tissue Remodeling While Preserving Core Biological Functions

Tissue‐resident macrophages are derived from embryonic progenitors, including yolk sac‐derived progenitors and fetal monocytes, and acquire distinct tissue‐specific programs by microenvironmental imprinting [51, 57]. In line with this, we observed that replaced macrophages acquired a distinct transcriptional profile from resident macrophages. For example, donor‐derived macrophages in the liver consistently exhibited lower levels of CD163 and TIM4. ATAC‐seq data further revealed that macrophages in the liver after Mr BMT retained core macrophage chromatin accessibility patterns while they did not fully establish the mature KC‐specific chromatin landscape. This result indicates that the developmental ontogeny shapes the characteristics of peripheral macrophages. In addition, we found that BMT caused long‐term remodeling in a tissue‐specific manner. A subset of genes, including *Cdkn1a*, *Phlda3*, *Psrc1*, and *Gtse1*, remained upregulated even after 9 months of transplantation, which suggests a sustained low‐grade stress adaptation after transplantation [30, 31, 32, 33]. Despite the transcriptional remodeling, the tissue integrity and core biological functions were largely preserved after tBMT and Mr BMT. Peripheral organs after tBMT and Mr BMT showed rapid and robust inflammatory responses after LPS challenge, characterized by the activation of innate immune pathways, canonical NF‐κB signaling, and inflammatory cytokine production. The overall pattern of these responses was generally similar to those observed in naïve mice. Taken together, our results suggest that even though BMT induces the remodeling of macrophage and tissue state, these alterations do not compromise tissue homeostasis or fundamental biological functions.

Overall, Mr BMT represents a promising cell therapy strategy for a broad spectrum of diseases, such as neurodegenerative disease [9, 10, 58, 59] and lysosomal storage disorder [11]. The clinical potential of microglial replacement has been preliminarily supported by our recent finding, which demonstrated effective disease arrest in both mouse models and human ALSP patients following Mr BMT [9]. Our current finding further suggest Mr BMT achieves durable systemic macrophages replacement while preserving tissue homeostasis, supporting its safety for future clinical application.

## 4 Methods and Materials

### 4.1 Animals

Cx3cr1‐GFP (B6.129P2(Cg)‐Cx3cr1^tm1Litt^/J, Strain #: 005582) [60] and β‐actin‐GFP mice (C57BL/6‐Tg (CAG‐EGFP)131Osb/LeySopJ, Strain#: 006567) [61] mice were obtained from The Jackson Laboratory (USA), while C57BL/6J mice were purchased from Charles River (Shanghai, China). All mice were housed under a 12‐h light/dark cycle with ad libitum access to food and water in the specific pathogen‐free (SPF) facility at Fudan University. All procedures were approved by the Institutional Animal Care and Use Committee of Fudan University (202110005S and 2025‐GZRJZ‐018).

### 4.2 Drug Administration

Mice were fed a PLX5622‐formulated AIN‐76A diet (1.2 g PLX5622 per kilogram of diet, formulated by SYSE Bio) ad libitum to pharmacologically deplete microglia and peripheral tissue‐resident macrophages. For depletion time‐course experiments, mice received PLX5622 chow for 7, 14, or 28 days. For Mr BMT, PLX5622 chow was administered for 14 days before myeloablative conditioning and was discontinued after transplantation unless otherwise indicated. Mice were treated with LPS (0.5 mg/kg body weight, Cat# L4391, Sigma–Aldrich) by intraperitoneal injection to elicit an immune response. Then, the mice were euthanized 3 h after administration.

### 4.3 Blood Collection and Biochemical Analysis

Mice were anesthetized, and whole blood was collected via cardiac puncture using a syringe pre‐rinsed with heparin sodium salt (Cat# H810907, Macklin) to prevent coagulation. Blood samples were immediately transferred into anticoagulant tubes and maintained at 4°C. Samples were centrifuged at 1000 × g for 10 min at 4°C, and the supernatant (plasma) was collected for subsequent biochemical analysis. Plasma biochemical parameters were measured, including liver function markers: alkaline phosphatase (ALP), alanine aminotransferase (ALT), aspartate aminotransferase (AST), total protein (TP), albumin (ALB), and cholinesterase (CHE); kidney function markers: urea nitrogen (UN); lipid metabolism markers: direct high‐density lipoprotein cholesterol (DHDL), triglycerides (TRIG), and total cholesterol (CHOL); cardiovascular‐related markers: creatine kinase (CK) and lactate dehydrogenase (LDH); and glucose metabolism marker: glucose (GLUH).

### 4.4 Tissue Preparation for the Cryosection and Immunohistochemistry

Mice were deeply anesthetized with isoflurane and transcardially perfused with 50 mL of 0.01 M cold PBS. Next, mice were perfused with 4% cold paraformaldehyde (PFA) for fixation. Following perfusion, brain and peripheral organs were dissected and subsequently postfixed in 4% PFA overnight at 4°C. All tissues were dehydrated in 30% sucrose in 0.01 M PBS at 4°C for one week and then embedded in optimal cutting temperature compound (OCT). Brain, liver, kidney, and spleen with regions of interest were sectioned using Leica CM1950 cryostat at a thickness of 35 µm, lung with regions of interest was sectioned at a thickness of 15 µm.

### 4.5 Immunohistochemistry and Image Acquisition

Brain and peripheral tissues sections were washed three times in 0.01 M PBS, followed by blocking and permeabilization in PBS containing 0.03% Triton X‐100 and 4% normal donkey serum (NDS, Cat# 017‐000‐121, Jackson) for 2 h at room temperature (RT). Sections were incubated overnight at 4°C with primary antibodies diluted in 1% NDS in PBST, followed by three times of wash with PBS. Secondary antibodies conjugated to fluorophores were applied for 2 h at RT in 1% NDS/PBST together with DAPI (1:1000, Cat# D9542‐10MG, Sigma–Aldrich). After washed three times with 0.03% PBST, samples were mounted in anti‐fade medium and sealed with nail enamel.

Primary antibodies information and concentration: rabbit anti‐IBA1 (1:500, Cat# 019‐19741, Wako); goat anti‐IBA1 (1:500, Cat# ab5076, Abcam); goat anti‐GFP (1:500, Cat# ab6673, Abcam); rabbit anti‐GFP (1:500, Cat# A‐11122, Thermo Fisher); rat anti‐F4/80 (1:500, Cat# 123101, Biolegend); rat anti CD68 (1:500, Cat# MCA1957, BIO‐RAD); mouse anti‐CD11c (1:500, Cat# 117314, Biolegend); rat anti‐CD163 (1:500, Cat# 14‐1631‐82, Thermo Fisher). Secondary antibodies information and concentration: donkey anti‐rat 647 (1:1000, Cat# A48272, Invitrogen); donkey anti‐rat 568 (1:1000, Cat# ab175475, Abcam); donkey anti‐rat 488 (1:1000, Cat# A21208, Thermo Fisher); donkey anti‐rabbit 488 (1:1000, Cat# 711‐545‐152, Jackson); donkey anti‐goat 488 (1:1000, Cat# 705‐545‐003, Jackson); donkey anti‐rabbit 568 (1:1000, Cat# A10042, Thermo Fisher); donkey anti‐rabbit 647 (1:1000, Cat# 711‐605‐152, Jackson).

Confocal images were captured using an Olympus FV3000 microscope equipped with solid‐state lasers (405, 488, 561, and 640 nm). Some whole‐brain fluorescence images were captured with an Olympus VS120 and VS200 microscope equipped with a motorized stage, using a ×10 objective lens. Z‐stack images were processed by maximum‐intensity projection in Fiji (ImageJ), with brightness and contrast adjusted when necessary.

### 4.6 Paraffin Sectioning and Hematoxylin and Eosin (H &E) Staining

Liver and lung tissues were collected and fully fixed in 4% paraformaldehyde (PFA) at 4°C. Following fixation, tissue samples were dehydrated in graded ethanol, paraffin‐embedded, and sectioned at a thickness of 5 µm. Tissue sections were deparaffinized in xylene, rehydrated through a graded ethanol series, and stained using a H &E staining kit (Cat# G1121, Solarbio) according to the manufacturer's instructions. After staining, sections were dehydrated, mounted, and imaged by a light microscope.

### 4.7 Bone Marrow Cells Preparation

Bone marrow cells were collected from adult Cx3cr1^+/GFP^ or β‐actin‐GFP mice. Briefly, femurs and tibias were harvested, cleared of surrounding muscle tissue, and rinsed twice in cold 0.01 M DPBS. Bone marrow cells were flushed out using a 10 mL syringe with a 26‐gauge needle. Bone marrow cells were centrifuged at 300 × g for 10 min. Pellets were then resuspended in PBS, filtered through a 40 µm cell strainer, counted, and diluted to appropriate concentrations for subsequent use.

### 4.8 Traditional Bone Marrow Transplantation (tBMT)

The tBMT procedure was performed as previously described with minor modifications [47]. In brief, adult C57BL/6J recipient mice were first fed with acidified water (pH 2–3) supplemented with neomycin (1.1 g/L) for 14 days. For irradiation‐based conditioning, recipient mice were exposed to a single dose of 9 Gy whole‐body X‐ray irradiation on day 14, followed by intravenous injection of 1 × 10^7^ GFP‐labelled bone marrow cells within 4–6 h after irradiation. For busulfan‐based conditioning, recipient mice received intraperitoneal busulfan at 25 mg/kg per day for four consecutive days from day 9 to day 12, with a cumulative dose of 100 mg/kg. Bone marrow cells were transplanted by tail‐vein injection on day 14, two days after the final busulfan injection.

### 4.9 Microglia Replacement by Bone Marrow Transplantation (Mr BMT)

The Mr BMT procedure was performed as previously described with minor modifications [7]. In brief, adult recipient C57BL/6J mice were fed with PLX5622‐formulated chow for 2 weeks in combination with acidified water (pH 2–3) and neomycin (1.1 g/L), followed by either 9 Gy whole‐body irradiation or intraperitoneal injection of busulfan (25 mg/kg body weight per day) for four consecutive days as myeloablative conditioning as previously described in tBMT.

Specifically, we quantified both GFP^+^IBA1^+^ donor‐derived and GFP^−^IBA1^+^ resident macrophages. The replacement efficiency/ratio was defined as the proportion of donor‐derived GFP^+^IBA1^+^ cells over total IBA1^+^ macrophages.

### 4.10 Preparation of the Liver and Lung Single‐Cell Suspension

Mice were deeply anesthetized with isoflurane and transcardially perfused with 50 mL of 0.01 M cold PBS. The liver and lung were harvested, mechanically minced into small pieces before being transferred into C‐tubes (Cat# 130‐093‐237, Miltenyi) containing digestion buffer, consisted of DMEM (Cat# C11995500BT, Gibco) supplemented with 100 U/mL DNase I (Cat# A510099, Sangon Biotech), 20 U/mL papain (Cat# LS003126, Worthington), 1 U/mL Dispase (Cat# D4693, Sigma), and 1.5 mg/mL trypsin inhibitor (Cat# 10109878001, Roche). Tissues were then dissociated through a gentle tissue dissociator (gentleMACS Octo, Miltenyi). Liver samples were processed using program 37C_m_LIDK_1, whereas lung samples were processed using program 37C_m_LDK_1. After enzymatic digestion and mechanical dissociation, cell suspensions were filtered through a 100 µm cell strainer to remove undigested tissue fragments. Cells were then centrifuged at 370 × g for 5 min at 4°C, and the cell pellets were resuspended for downstream analyses.

### 4.11 Fluorescence‐Activated Cell Sorting (FACS)

Liver single‐cell suspension was prepared from 11.5 month‐old naïve and Mr BMT mice. For ATAC‐seq, the cells were stained with anti‐CD45 antibody (1:100, clone 30‐F11, Cat# 103105, Biolegend) for 30 min in the dark at 4°C, and dead cells were excluded by DAPI (1:2000, Cat# 564907, BD Pharmingen). Living CD45^+^ cells (CD45^+^DAPI^−^) were sorted by a FACSAria III cell sorter (BD Biosciences). ATAC‐seq libraries were subsequently generated using the Hyperactive ATAC‐Seq Library Prep Kit for Illumina (Cat# TD711‐01, Vazyme) according to the manufacturer's instructions. For bulk RNA‐seq, liver single‐cell suspensions were prepared from 11.5 month‐old naïve and Mr BMT mice. The cells were stained with antibodies against CD45 (1:100, clone 30‐F11, Cat# 103105, Biolegend) and F4/80 (1:100, clone T45‐2342, Cat# 566787, BD Pharmingen) for 30 min in the dark at 4°C. After exclusion of dead cells by DAPI staining, live macrophages (DAPI^−^CD45^+^F4/80^+^) cells were sorted using a FACSAria III cell sorter (BD Biosciences) and subjected to bulk RNA‐seq analysis.

### 4.12 Flow Cytometry Analysis

Single‐cell suspensions from liver and lung were prepared as described above and stained with fluorophore‐conjugated antibodies against CD11b (1:100, clone M1/70, Cat# 553312, BD Pharmingen, or Cat# 101207, Biolegend), CD45 (1:100, clone 30‐F11, Cat# 563709, BD Pharmingen), Tim4 (1:100, clone RMT4‐54, Cat# 12‐5866‐82, Thermo Fisher), F4/80 (1:100, clone BM8, Cat# 123113, Biolegend), CD11c (1:100, clone N418, Cat# 117339, Biolegend) for 30 min in the dark at 4°C. After that, cells were washed and resuspended in staining buffer with DAPI (1:2000, Cat# 564907, BD Pharmingen) for acquisition. Flow cytometric data were collected by LSRFortessa (BD Biosciences) and processed by FlowJo 10 (BD Biosciences).

### 4.13 RNA Extraction and Quantitative Polymerase Chain Reaction (qPCR) Analysis

Total RNA was isolated from peripheral organs using the RNA extraction kit (Cat# 9767, Takara). Then, the extracted RNA was reverse‐transcribed into cDNA with the RT super mix for qPCR (Cat# R323‐01, Vazyme) according to the manufacturer's instructions. Quantitative PCR was performed with one‐step SYBR qPCR master mix (Cat# Q711‐02, Vazyme). *Gapdh* was used as the internal control gene. Primers used in this study are listed in Table S1.

For qPCR analysis, raw expression matrices from the liver, kidney, lung, and spleen were imported into R (v4.3.2). Relative gene expression changes were calculated and visualized as log_2_ fold changes.

### 4.14 ELISA

Serum levels of IL‐6, TNF‐α, IL‐1β and IFN‐γ were measured using commercial ELISA kits according to the manufacturer's instructions, including IL‐6 (Cat# KE10007, Proteintech), TNF‐α (Cat# KE10002, Proteintech), IL‐1β (Cat# KE10003, Proteintech) and IFN‐γ (Cat# KE10094, Proteintech). Total protein concentrations were determined by BCA protein assay kit (Cat# P0012, Beyotime). Cytokine concentrations were calculated from standard curves generated using absorbance values measured at 450 nm and were normalized to total protein concentrations.

### 4.15 RNA‐seq Data Processing and Quantification

Raw RNA‐seq data were processed using Trimmomatic v0.39 in paired‐end mode to remove sequencing adapters, and reads shorter than 36 bp after trimming were discarded. The remaining clean reads were mapped to the GRCm39 reference genome using HISAT2 v2.1.0. Aligned reads were subsequently sorted with SAMtools v1.10, and gene‐level read counts were quantified using featureCounts v2.1.1 with the GRCm39 GTF annotation. For downstream analyses, FPKM values were normalized by adding a pseudo‐count of 1 followed by log2 transformation. Genes with zero variance across all samples were removed. Sample correlations were computed using the cor function in R, and principal component analysis (PCA) was performed on the filtered expression matrix using the prcomp function. PCA results were visualized with factoextra v1.0.7. Differential expression genes were identified using DESeq2 v1.42.0. Genes were considered significantly differentially expressed when they met the criteria of an adjusted *p*‐value (padj) < 0.05 and an absolute log2 fold change > 1. GO and KEGG pathway enrichment analyses were performed using clusterProfiler v4.12.6; (enrichGO and enrichKEGG functions), with categories having a false discovery rate (FDR) ≤ 0.05 considered significantly enriched. Enrichment results were visualized using dotplot and emapplot, and Venn diagrams, heatmaps, and volcano plots were generated using VennDiagram v1.7.3, heatmap v1.0.12, and ggplot2 v3.5.1, respectively.

### 4.16 Immune Cell Deconvolution Analysis

Immune cell composition was estimated using the CIBERSORT algorithm, a machine learning deconvolution approach based on linear support vector regression (SVR) for quantifying 22 immune cell subtypes from bulk RNA‐seq data [62]. CIBERSORT analysis was implemented using the IOBR 0.99.0 package.

### 4.17 ATAC‐seq Data Processing and Analysis

ATAC‐seq libraries were generated and sequenced for the indicated samples. Initially, raw sequencing reads were assessed using FastQC (v0.12.1) to evaluate sequencing quality. Adapter trimming and low‐quality read filtering were performed using fastp (v0.24.1) with paired‐end adapter auto‐detection enabled. Clean reads were then aligned to the mouse reference genome (mm10) using Bowtie2 (v2.5.4) with the parameters “–very sensitive”, “–dovetail”, and a maximum fragment length of 1000 bp. SAM/BAM processing, sorting, indexing, and mapping statistics were performed using SAMtools (v1.21). To improve data quality, mitochondrial reads (chrM) were removed, and read group information was added using Picard (v2.20.4). PCR duplicates were identified and marked using the Picard MarkDuplicates function. Properly paired reads with mapping quality scores ≥ 30 were retained for downstream analysis using SAMtools with the parameters “‐f2 ‐F 1548 ‐q 30”. Reads overlapping ENCODE blacklist regions were removed using BEDTools (v2.31.1) and the mm10 blacklist annotation. To correct for Tn5 transposase insertion bias, filtered BAM files were adjusted using the “‐ATACshift” function implemented in deepTools (v3.5.6) alignmentSieve. The shifted BAM files were subsequently sorted and indexed, and normalized genome coverage tracks were generated using deepTools bamCoverage with BPM normalization and a bin size of 10 bp. For downstream chromatin accessibility analysis, peak calling was performed using MACS2 (v2.2.9.1). Differentially accessible chromatin regions between experimental groups were identified using appropriate statistical approaches. Peak annotation to genomic regions and nearby genes was conducted using the R package ChIPseeker (v1.44.0). Functional enrichment analysis of peak‐associated genes was performed using the R package clusterProfiler (v4.16.0). Transcription factor motif enrichment analysis was conducted using the HOMER utility findMotifsGenome.pl.

### 4.18 Transcription Factor Occupancy Analysis

Transcription factor occupancy was inferred from ATAC‐seq data using TOBIAS (v0.16.1). Briefly, Tn5 insertion bias was corrected using the ATACorrect function, footprint scores were calculated using ScoreBigwig, and differential transcription factor occupancy between groups was analyzed using BINDetect based on merged accessible chromatin regions and transcription factor motif databases. Aggregate footprint plots and footprint heatmaps were generated using the PlotAggregate and PlotHeatmap functions, respectively.

### 4.19 Behavioral Tests

Behavioral tests were performed during the light phase by investigators blinded to group allocation. Mice were habituated to the testing room for at least 30 min before each test. Apparatuses were cleaned with 75% ethanol between trials. The order of behavioral assays was arranged from less stressful to more stressful tests with at least 24 h between tests.

### 4.20 Y Maze Test

Spontaneous alternation was evaluated using a Y‐shaped maze composed of three arms (30 cm × 30 cm × 5 cm) at 120° angles from each other. The Y maze was placed on the ground, and the mouse behavior was recorded with an overhead camera. Mice were placed at the end of one arm and allowed to freely explore the maze for 10 min. Behavioral tracking and analysis were performed using EthoVision 11.5.1022 (Noldus). Spontaneous alternation performance was expressed as the percentage of correct alternations, calculated as: correct alternation rate (%) = number of correct alternations/total possible alternations × 100%. Total possible alternations were defined as the total number of arm entries minus two.

### 4.21 Open Field Test

Individual mice were placed in an open field area (40 cm × 40 cm) and allowed to explore for 10 min freely. Anxiety‐related behavior was assessed by measuring the time spent in the center (center of the area, 20 cm × 20 cm) using EthoVision 11.5.1022 (Noldus).

### 4.22 Novel Object Recognition Test (NOR)

The novel object recognition test was performed with minor modifications based on our previous protocol [26]. Briefly, a 40 cm × 40 cm open field box was used throughout the experimental apparatus. On day 1, mice were habituated to the apparatus for free exploration for 10 min (pre‐training, which also worked as an open field test). On day 2, the mice were familiarized with the objects for 30 min, and the mice were allowed to freely explore the apparatus with two identical objects for 3 min (stage I) and then were put back in the home cage. After a 5‐min interval, one familiar object was replaced with a novel object. The mouse explored the open field with one familiar object and one novel object for 5 min. Times spent on each object were analyzed by EthoVision 11.5.1022 (Noldus). The recognition index = time of novel object exploration / (time of novel object exploration + time of familiar object exploration).

### 4.23 Elevated Plus Maze

Anxiety‐like behavior was evaluated using an elevated plus maze with two open arms and two closed arms (arms 30 × 5 cm, with side walls 15 cm high on two closed arms, elevated 50 cm above the ground). Mice were individually placed in the center of the maze and allowed to explore for 5 min. The time spent in the open arms was quantified using EthoVision 11.5.1022 (Noldus).

### 4.24 Rotarod

For the rotarod test, mice were placed on an accelerating rotarod cylinder, the speed was slowly accelerated from 4 to 40 rpm over 5 min and then maintained at 40 rpm for an additional 5 min. A trial ended when the animal dropped or clung to the rod and completed three passive rotations. The latency to fall was recorded. Data were presented as the mean of the average time to drop across three trials.

### 4.25 Metabolic Cage Analysis

Mice were performed metabolic cage analysis at 1‐ or 9‐month post tBMT, and Mr BMT. Metabolic parameters were assessed using a metabolic monitoring system (CLAMS, USA). Mice were individually housed in metabolic cages and acclimated to the system for 48 h before data collection. After acclimation, metabolic parameters were continuously recorded for 24 h under a 12 h light/12 h dark cycle with free access to food and water. Oxygen consumption (VO_2_), carbon dioxide production (VCO_2_), respiratory exchange ratio (RER = VCO_2_/VO_2_), energy expenditure (EE), locomotor activity (total X‐Axis activity and Ambulatory X‐Axis activity) was monitored automatically. EE values were normalized to body weight when indicated.

### 4.26 Statistical Analysis

Statistical analyses were conducted using Prism 10 (GraphPad). Each data point represented the mean value obtained from three sections in different regions of the brain, liver, kidney, lung, and spleen. Data were presented as mean ± standard deviation (SD). Unpaired or paired t‐tests were used for comparisons between two groups, as appropriate. One‐way analysis of variance (ANOVA) followed by Tukey's multiple comparisons test or the nonparametric Kruskal–Wallis test was used for comparisons among multiple groups. Pairwise Wilcoxon rank‐sum tests were used for nonparametric pairwise comparisons, unless otherwise stated. Statistical significance was defined as *p* < 0.05.

Results were determined independently in a double‐blind manner. No statistical methods were used to predetermine sample sizes. All mice were randomly divided into different groups for the experiment. Experiments were performed in at least two independent batches to reduce potential batch effects. The data distribution was assumed to be normal, but this assumption was not formally tested. No data were excluded from the analyses.

## Author Contributions

Yanxia Rao supervised and conceptualized this study. Yanxia Rao, Bo Peng, Yufei Xu, Miaozhan Zou, Zhihao Jin, Baozhi Yang, and Yang He wrote the manuscript. Yufei Xu, Miaozhan Zou, and Yunshang Bai performed most of the experiments and analyzed the data; Jinjin Du, Pei Ouyang, Yuanyuan Cai, Yuheng Zhang, Bingying Du, Chengcheng Ge, Jian‐an Gong, and Xiaoyu Li participated in other experiments; Zhihao Jin, Miaozhan Zou, and Yufei Xu analyzed bulk RNA‐seq and ATAC‐seq data. Baozhi Yang, Yuanyi Zheng, and Jing Ding provided necessary study support. All authors discussed the results and provided critical feedback on the manuscript.

## Ethics Statement

All animal experiments were conducted in accordance with the guidelines of the Institutional Animal Care and Use Committee of Laboratory Animal Center at Fudan University (permit number: 202110005S and 2025‐GZRJZ‐018).

## Conflicts of Interest

Yanxia Rao, Bo Peng, and Yuheng Zhang are applying for patents related to microglia replacement. Other authors declare no conflicts of interest.

## Supporting information




**Supporting File**: advs76763‐sup‐0001‐SuppMat.docx.

## Data Availability

All sequencing data generated in this study have been deposited in the NCBI Gene Expression Omnibus (GEO) database under accession numbers GSE334348 (tissue bulk RNA‐seq), GSE334349 (ATAC‐seq), and GSE334350 (sorted macrophage bulk RNA‐seq).
