# Supplementary material for: Mr BMT Achieves Systemic Macrophage Replacement With Preservation of Tissue Homeostasis
Source: Adv Sci (Weinh). 2026 Aug 14;13(58):e19669. doi: 10.1002/advs.202519669 (PMC13474184; doi:10.1002/advs.202519669)
Supplement: Supplementary file 1 [file ADVS-13-e19669-s001.docx]

**Supplementary Figures**

**Figure S1.** **Co-localization of IBA1 with F4/80 and CD68 in peripheral organs.**

(A–B) Representative confocal images (A) and quantification (B) of IBA1 (red) and F4/80 (green) co-localization in liver, kidney, lung, and spleen tissues from naïve mice.

(C–D) Representative confocal images (C) and quantification (D) of IBA1 (red) and CD68 (green) co-localization in liver, kidney, lung, and spleen tissues from naïve mice.

N = 6–10 biological replicates for each group. Data are presented as mean ± SD.

**Figure S2.** **PLX5622 systemically depletes microglia and macrophages across tissues.**

(A) Experimental design.

(B) Representative images show the depletion efficiency of microglia and macrophages in the brain and peripheral organs following the administration of PLX5622.

CD: control diet; D: day.

**Figure S3.** **Efficient replacement of tissue-resident macrophages in peripheral organs under busulfan conditioning.**

(A) Scheme of the experimental timeline for Mr BMT and tBMT under busulfan conditioning.

(B–C) Representative confocal images (B) and quantification (C) of BM-derived cells (GFP, green) and macrophages (F4/80, red) in liver, kidney, lung, and spleen after Mr BMT or tBMT.

(D–E) Representative confocal images (D) and quantification (E) of BM-derived cells (GFP, green) and macrophages (IBA1, magenta) in liver after Mr BMT or tBMT.

N = 4–5 biological replicates for each group. Data are presented as mean ± SD. Unpaired t-test was used for statistical analysis. P values are shown in the figures. CD, control diet; i.v., intravenous injection; D, day.

**Figure S4.** **Mr BMT elicits distinct immune signatures across peripheral organs, compared with the naïve group.**

(A-D) Heatmaps show selected DEGs in liver (A), kidney (B), lung (C), and spleen (D).

(E) GO enrichment showing the top 10 enriched signaling pathways across multiple tissues following Mr BMT.

**Figure S5. Persistent organ-specific transcriptional alterations across peripheral organs following Mr BMT.**

(A) Experimental design. Timepoint for Mr BMT in the scheme is defined as the time of BMC transplantation.

(B, D, F, H) Volcano plots showing DEGs in liver (B), lung (D), kidney (F), and spleen (H) from Mr BMT versus naïve mice at 3.5 and 11.5 MO.

(C, E, G, I) Venn diagrams showing overlapping upregulated and downregulated DEGs between the 3.5 MO and 11.5 MO time points in liver (C), lung (E), kidney (G), and spleen (I).

MO, month-old.

**Figure S6. Mr BMT mice retain robust immune response to LPS challenge.**

(A) Experimental timeline. Timepoint for tBMT and Mr BMT in the scheme is defined as the time of BMC transplantation.

(B–D) Heatmaps showing innate immune response-related genes measured by qPCR across multiple tissues in naïve (B), tBMT (C) and Mr BMT (D) following LPS treatment. Bar plots showing the expression of *Myd88* and *Cd14.*

(E) Experimental timeline.

(F) PCA of bulk RNA-seq datasets from liver, kidney, lung, and spleen tissues in naïve and Mr BMT groups after LPS treatment at 11.5 MO.

(G–J) Heatmaps showing overlapping LPS-responsive genes in liver (G), kidney (H), lung (I), and spleen (J) from naïve and Mr BMT mice.

N = 4 biological replicates for each group. Data are presented as mean ± SD. Unpaired t-test was used for statistical analysis. P values are shown in the figures. MO, month-old.

**Figure S7. Characteristics of donor-derived macrophage in liver and lung following Mr BMT.**

(A–B) Representative confocal images (A) and quantification (B) of donor-derived cells (GFP, green), macrophages (IBA1, red), and CD163 (gray) in liver from naïve, tBMT, and Mr BMT groups at 3.5 MO.

(C–E) Representative flow cytometric plots (C) and quantification of lung macrophage populations (D–E) in naïve, tBMT, and Mr BMT groups at 3.5 MO. Alveolar macrophages (AMs) and interstitial macrophages (IMs) were identified based on CD11c and CD11b expression.

N = 5–6 biological replicates for each group. Data are presented as mean ± SD. Unpaired t-test was used for comparison between the tBMT and Mr BMT groups, while One-way ANOVA followed by Tukey’s multiple comparisons test was used for the comparisons among naïve, tBMT and Mr BMT groups. P values are shown in the figures. MO, month-old.

**Figure S8. Mr BMT preserve long-term systemic biochemical homeostasis.**

(A) Experimental design. Timepoint for tBMT and Mr BMT in the scheme is defined as the time of BMC transplantation.

(B) Quantification of serum biochemical parameters in naïve, tBMT, and Mr BMT groups at 3.5 MO.

(C) Experimental design.

(D) Quantification of serum biochemical parameters in naïve and Mr BMT groups treated with PBS or LPS at 11.5 MO.

N = 4 biological replicates for LPS treatment group, and N = 6 biological replicates for PBS treatment group. Data are presented as mean ± SD. One-way ANOVA followed by Tukey’s multiple comparisons test was used for statistical analysis. P values are shown in the figures. MO, month-old.

| Primers used in the experiment | | |
| --- | --- | --- |
| Genes | Forward | Reverse |
| *Il1b* | TGGACCTTCCAGGATGAGGACA | GTTCATCTCGGAGCCTGTAGTG |
| *Cd74* | AGATGCGGATGGCTACTCC | TCATGTTGCCGTACTTGGTAAC |
| *Gapdh* | CATGGCCTTCCGTGTTCCTA | CCTGCTTCACCACCTTCTTGAT |
| *Ccl5* | GCTGCTTTGCCTACCTCTCC | TCGAGTGACAAACACGACTGC |
| *Socs3* | ATGGTCACCCACAGCAAGTTT | GCTCCAGTAGAATCCGCTCTC |
| *Ccl3* | CTCCCAGCCAGGTGTCATTTT | CTTGGACCCAGGTCTCTTTGG |
| *Cd14* | ACTTCTCAGATCCGAAGCCAG | CCGCCGTACAATTCCACAT |
| *Myd88* | TCATGTTCTCCATACCCTTGGT | AAACTGCGAGTGGGGTCAG |
| *Il6* | CTGCAAGAGACTTCCATCCAG | AGTGGTATAGACAGGTCTGTTGG |
| *Tnf* | CTGAACTTCGGGGTGATCGG | GGCTTGTCACTCGAATTTTGAGA |
| *Cxcl13* | GGCCACGGTATTCTGGAAGC | GGGCGTAACTTGAATCCGATCTA |
| *Il10* | GCCAGAGCCACATGCTCCTA | GATAAGGCTTGGCAACCCAAGTAA |

**Table S1.** Primers used in this study.
